# Care and support networks of community-dwelling frail individuals in North West London: a comparison of patient and healthcare workers’ perceptions

**DOI:** 10.1186/s12877-022-03561-y

**Published:** 2022-12-09

**Authors:** David Sunkersing, Finbarr C. Martin, Paul Sullivan, Derek Bell

**Affiliations:** 1 Department of Primary Care and Public Health, Imperial College London, London, W6 8RP UK; 2 King’s College London (Population Health Sciences), London, UK

**Keywords:** Community-Dwelling, Older People, Frailty, Care and Support Networks, Social network analysis

## Abstract

**Background:**

Evidence suggests that successful assessment and care for frail individuals requires integrated and collaborative care and support across and within settings. Understanding the care and support networks of a frail individual could therefore prove useful in understanding need and designing support. This study explored the care and support networks of community-dwelling older people accessing a falls prevention service as a marker of likely frailty, by describing and comparing the individuals’ networks as perceived by themselves and as perceived by healthcare providers involved in their care.

**Methods:**

A convenience sample of 16 patients and 16 associated healthcare professionals were recruited from a community-based NHS ‘Falls Group’ programme within North-West London. Individual (i.e., one on one) semi-structured interviews were conducted to establish an individual’s perceived network. Principles of quantitative social network analysis (SNA) helped identify the structural characteristics of the networks; qualitative SNA and a thematic analysis aided data interpretation.

**Results:**

All reported care and support networks showed a high contribution level from family and friends and healthcare professionals. In patient-reported networks, ‘contribution level’ was often related to the ‘frequency’ and ‘helpfulness’ of interaction. In healthcare professional reported networks, the reported frequency of interaction as detailed in patient records was used to ascertain ‘contribution level’.

**Conclusion:**

This study emphasises the importance of the role of informal carers and friends along with healthcare professionals in the care of individuals living with frailty. There was congruence in the makeup of ‘patient’ and ‘provider’ reported networks, but more prominence of helper/carers in patients’ reports. These findings also highlight the multidisciplinary makeup of a care and support network, which could be targeted by healthcare professionals to support the care of frail individuals.

**Supplementary Information:**

The online version contains supplementary material available at 10.1186/s12877-022-03561-y.

## Background

Frailty is a clinical syndrome, commoner in older adults, and associated with an increased risk of adverse health outcomes including falls, hospitalisation and reduced survival time [1–3]. As older adults living with frailty are more likely to experience or develop disability and to experience reduced self-efficacy, their care and support networks may be of increased benefit to them. In the UK, support for frail individuals living in the community is usually provided in two main ways: by family, friends or neighbours without payment (informal care), or through services they or their local authority pay for (formal care) [4]. An individual may also decide to receive care and support from a combination of these [4].

Many frail individuals rely on family, friends, groups, healthcare professionals, carers and others to assist with their care and support needs and maintain both their independence and quality of life (a care and support network) [5]. Despite this, many frail individuals do not receive the care and support they need [6]. Having a care and support network is important, as evidence suggests that more socially connected adults are healthier and live longer than more isolated peers [7–9]. Whilst the individuals making up the care and support network share a focal point – the frail individual – they may comprise multiple disciplines [10]. Understanding a frail individual’s care and support network from their perspective as well as the healthcare professionals involved in their care could improve patient care and experience and open up opportunities for collaborative care and support. This could be through better understanding of the services, local authorities or people identified (or available) in the care and support networks, to help with care planning, and transfers of care (e.g., from a hospital setting into a community setting) [11, 12].

Research has noted the difficulty in verbally describing an individual’s social networks, but that a network visualisation (where an individual’s connections are mapped) can aid in communicating the social network [13, 14]. It therefore might be useful to care providers if a care and support network could be encapsulated (i.e., visually) and communicated between them and subsequently to others at times of transitions of care. To our knowledge, few studies have reported examining community-dwelling frail individuals in the UK considering both ‘patient’ and provider perspectives using a network approach.

Frailty is a complex multidimensional condition. There are various approaches to its identification and operationalisation in health and care settings [15, 16]. At the time of this study, there was no widely used systematic approach to identifying a population of older community dwelling people living with frailty. In this study, people presenting for healthcare because of a recent fall or identified to be at high risk of falling and receiving care from a falls prevention service were chosen as an indicator of a population with frailty, as many studies have found frailty to be an independent predictor of falls [17–22] and recommendations state that falls should be identified as a marker of frailty [23–25] with falls currently being used as a marker of frailty in practice [26–28]. Further, the primary author who interviewed the patients (DS) is not a clinician, which is a requisite to clinically identify an individual as frail using a clinical tool (e.g., the electronic Frailty Index (eFI) or Clinical Frailty Scale (CFS)) and/or to conduct a clinical examination to ascertain clinical frailty.

The aim of this study was to gain a greater insight into perceptions of patients and care providers regarding their care and support networks.

## Methods

### Study design

The research used non-experimental (no manipulation of human subjects), semi-structured interviews with an activity (explained below) to investigate the care and support networks of frail individuals using a community falls prevention service (FPS).

### Setting

An NHS community-based FPS in North West London, managed by the Central London Community Healthcare (CLCH) NHS Trust. The service is free and accessed by referral from any relevant local NHS or social care provider or occasionally self-referral.

### Participants

#### Patients

All consecutive patients having attended at least 2 FPS sessions were eligible to be approached and those giving initial willingness to consider participation were given a ‘Participant Information Sheet’.

Exclusion criteria were, lack of capacity to give informed consent (e.g., inability to understand the information sheet) or inadequate command of English to complete an individual (i.e., one on one) interview, as judged by the researcher or healthcare provider.

All patient participants gave written informed consent (Additional file 1: Appendices A—D).

#### Healthcare professionals

These participants also received a participant information sheet and agreed to be interviewed about each patient participant.

All healthcare professional participants gave written informed consent (Additional file 1: Appendices A—D).

### The questionnaire

The interview questions were initially tested at a general level (academics (4), clinicians (2), project managers (2), frail individuals (2) and with 13 healthcare professionals (consisting of 1 × Clinical Lead, 1 × Consultant Physiotherapist, 2 × Physiotherapists, 1 × Nurse, 3 × Occupational Therapists, 4 × Rehabilitation/Physiotherapist Assistants, 1 × Support Worker) for purpose and clarity to ensure that they would be suitable and understood by participants. After testing, the feedback was analysed, and the questions were revised (Additional file 1: Appendices E-F).

### Data collection

Each semi-structured interview was completed and conducted at the site of the FPS, ensuring completion in a familiar environment. Interviews were audio-recorded and transcribed. The activity component involved the researcher (DS) conducting the interview, then arranging the interview responses on Post-it notes and placing them on a ‘Concentric Circle of Influence’ diagram to help facilitate discussion with each participant (patient or healthcare professional), allow responses to be checked, changed and sorted. This activity was similar to that published in previous studies [29], and comprises two stages:

### Name generation

a) Patients were asked to identify (by work role, not name) the individuals/professions involved in their care and support (Additional file 1: Appendix E). Participants could mention anyone they believed was involved in their care and support (e.g., not restricted to social or healthcare providers). As each individual/profession was generated, it was written on a Post-it note.

### Contribution level (‘Concentric Circle of Influence’ diagram)

**Fig. 1:**
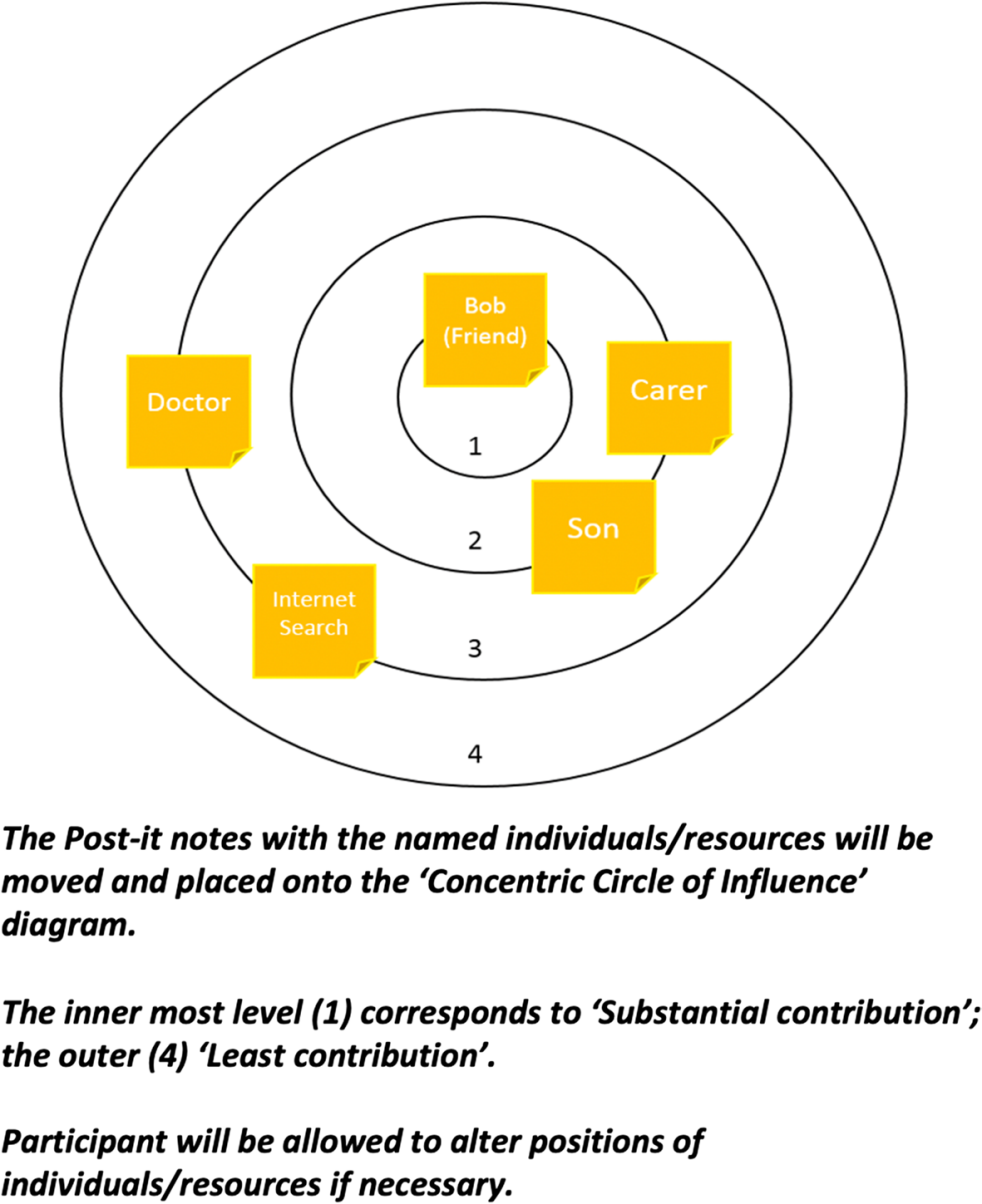
Contribution Level

a) Contribution level was ascertained by asking patients how much they believed the reported individual(s) contributed towards their care and support. Participants were asked to signify the level of contribution, with reference to four ‘contribution levels’ (Fig. 1). The Post-it notes with the named individuals/resources were then moved and placed onto a ‘Concentric Circle of Influence’ diagram (Fig. 1), the innermost level corresponding to ‘Substantial contribution’; the outer ‘Least contribution’. Each patient was able to alter the positions of individuals/resources as necessary throughout the course of the activity/interview. At this stage, a discussion with the patient took place to help understand why the named individual/resource was placed on a particular level (e.g., because of frequency). Interim analysis and data monitoring took place throughout the study (by DS) to identify when thematic saturation had been reached (for both groups), after which recruitment was concluded. Thematic saturation in this study referred to when no new individuals/professions were mentioned during interviews with the participants (e.g., the responses ‘Patient C’ gave were more or less identical to earlier interviews with ‘Patient B’ and ‘Patient A’) (i.e., qualitative) and when no new network patterns emerged in the rationale for an individual’s contribution level.

### Healthcare Professional (HCP) Interviews

The activity/interview questions were similarly structured but re-worded to determine the network of the patient (Additional file 1: Appendix F) as perceived by the HCP. Each HCP completed the ‘Concentric Circle of Influence’ diagram electronically for the individual patients they had direct contact with and were able to refer to patient notes. The concept of contribution was explained to the HCPs in the same way as described to the patients.

An ‘ego network’ placing the individual (the ego) at the centre of the network diagram surrounded by all the connections (ties) mentioned was created from the activity to visualise and help analyse the networks. The software program 'NetDraw’ [30] was used to draw and visualise these network diagrams electronically.

### Collating the networks

Each of the 16 patient perspective networks were collated into one network. The same was done for each of the 16 healthcare professional perspective networks. This was done to facilitate visual comparison between perspectives (i.e., both patient-patient network comparison and patient-healthcare professional network comparison).

### Data analysis

The network diagrams of the frail individuals were visually inspected for trends and distributions.

Qualitative data, derived from several questions during the activity were analysed using the principles of thematic analysis using cross-case comparisons [31]. Principles from Braun and Clarke’s framework [32] and others [33] were used to create defined themes. Qualitative aspects of the different perspectives were compared through thematic analysis. Content analysis was performed (by DS and cross-checked by DB, FM and JR), which included grouping categories into themes. The common themes arising from the participants were then examined and analysed with regards to the care and support received.

## Results

### Participants

All potential patient participants met the inclusion criteria. None met the exclusion criteria. 18/44 eligible patients (41%) completed a consent form and 16 patients participated in the study, mean age 72 years (range 64–81), 3 men and 13 women. As thematic saturation was reached for both patients and healthcare providers after 16 interviews for each had been conducted, not all eligible patients were interviewed.

Twelve patients had been referred to the FPS by their GP, 1 self-referral, 1 from their Podiatrist, 1 from Age UK and 1 from a hospital consultant.

Sixteen healthcare professionals (all physiotherapists from the FPS) participated, noting that they had based their judgement of contribution level on the reported frequency of interaction with the patient, as recorded in a patient’s notes.

### Quantitative and descriptive analyses

During the interviews, different individuals/activities (connections) were noted, which for visual and mathematical comparison, were grouped into 5 distinct categories. These were: Healthcare Professionals (persons associated with a speciality or discipline who are qualified and allowed by regulatory bodies to provide healthcare services to patients), Family/Friends (who provided care and support without a fee), Group Activities (e.g., dance classes), Carers/Helpers (individuals who were not Family/Friends, but were providing care and support in a voluntary capacity (often without formal training), e.g., via a charity) and Other (individuals mentioned that did not fit into the other four categories).

**Fig. 2:**
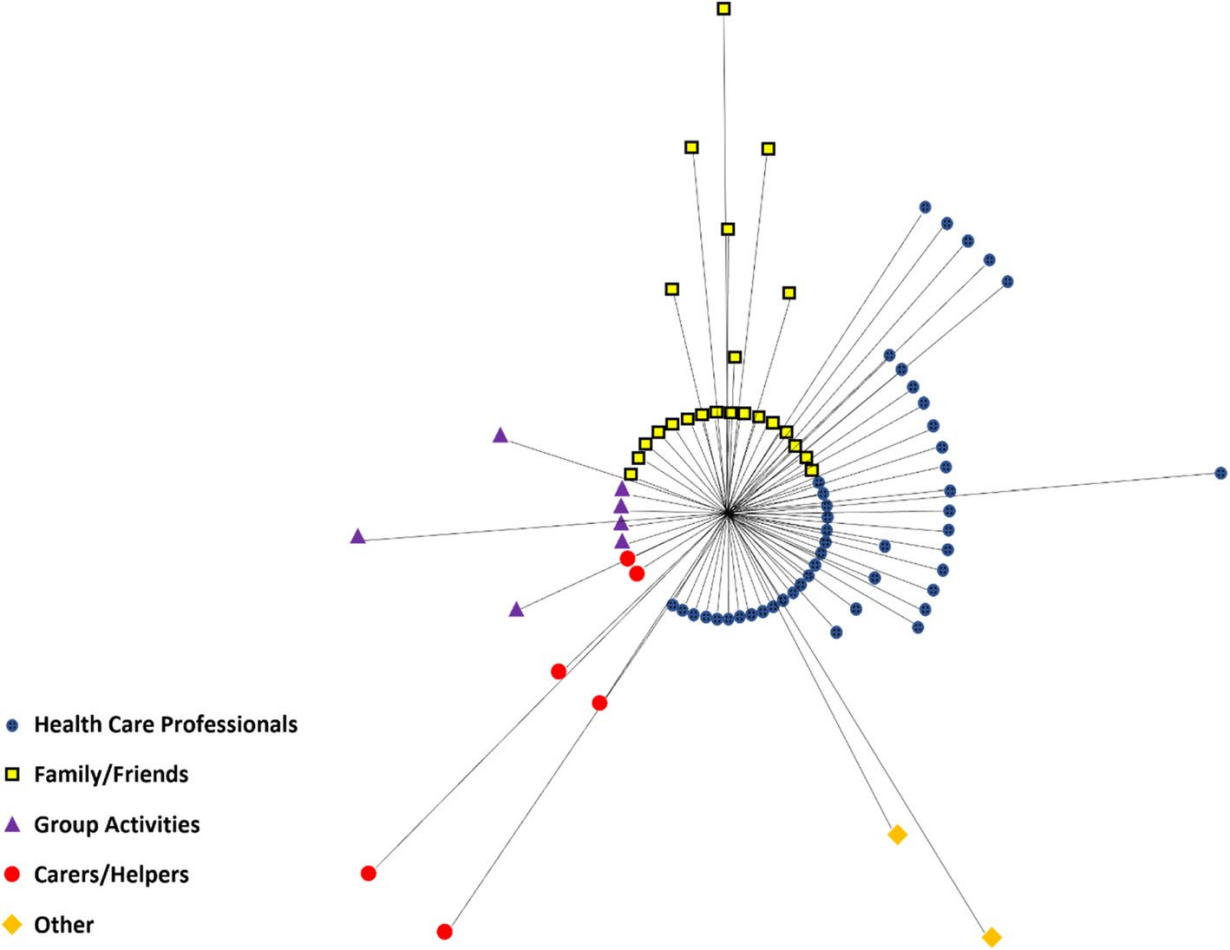
Collated Networks (Patient Reported). Lines indicate a link between ego (Patient) in the centre and a connection (Family/Friends, Health Care Professionals, Group Activities, Carers/Helpers or Other). The ‘Other’ category comprises individuals or services that do not fit into the other four categories. Shorter lines represent stronger ties, longer lines represent weaker ties (based on responses to ‘Contribution Level’)

**Fig. 3:**
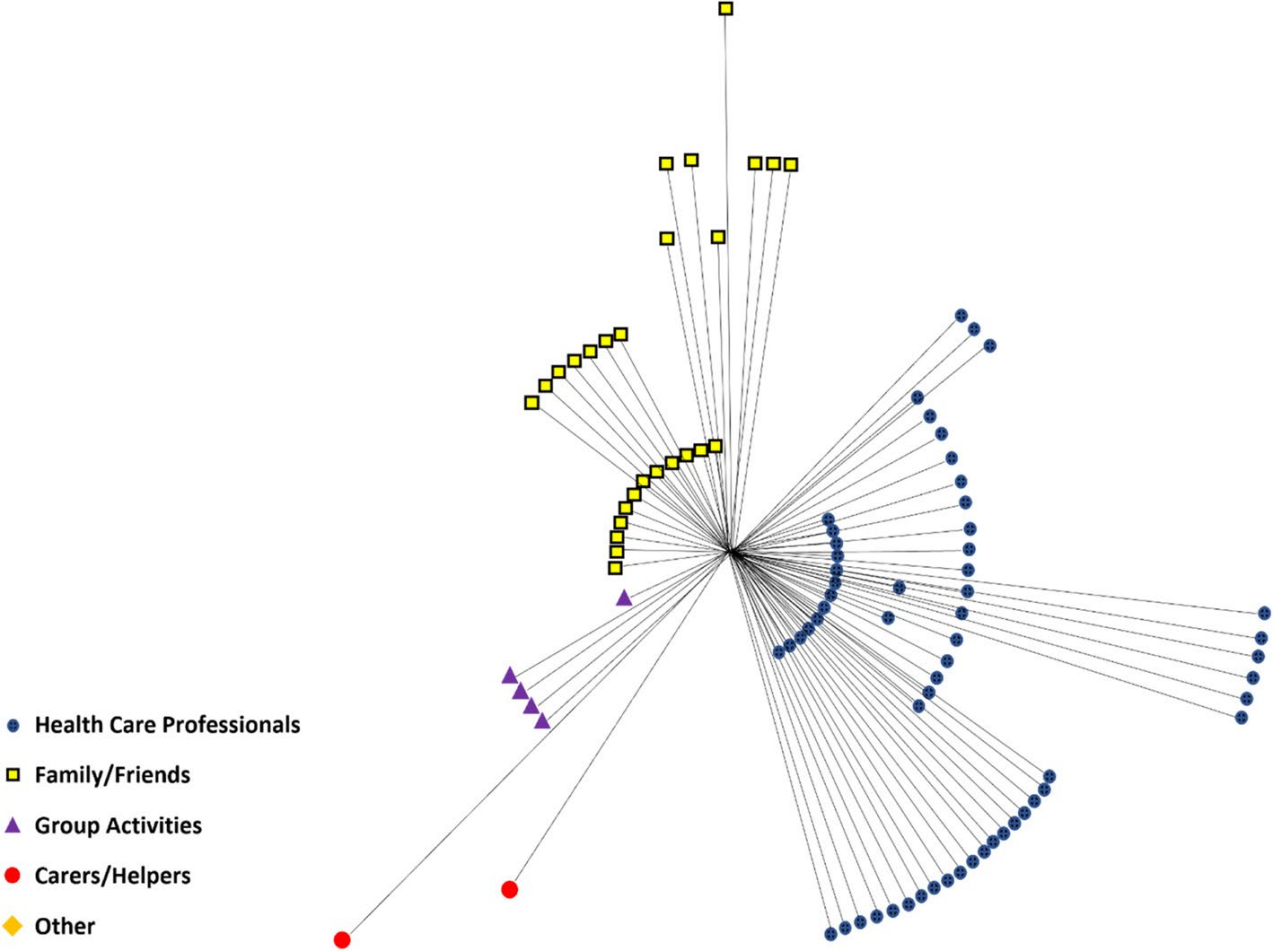
Collated Networks (HCP Reported). Lines indicate a link between ego (Patient) in the centre and a connection (Family/Friends, Health Care Professionals, Group Activities, Carers/Helpers or Other). The ‘Other’ category comprises individuals or services that do not fit into the other four categories. Shorter lines represent stronger ties, longer lines represent weaker ties (based on responses to ‘Contribution Level')

The biggest range in contribution levels (shown in Figs. 2 and 3) was evident in the ‘Family/Friends’ category in patient reported networks (contribution range: 1–4). For healthcare professional reported networks, it was evident in both the ‘Family/Friends’ and ‘Health Care Professionals’ category (contribution range: 1–4).

### Collating the networks

The 16 individual participants’ networks were collated into one ego-centric network (patient perspective, Fig. 2). The 16 networks reported from the healthcare professionals were collated into one ego-centric network (HCP perspective, Fig. 3).

### Qualitative reasoning behind choices: thematic analysis

Three overarching themes were identified: helpfulness of individual, personal contacts and frequency of contact (example quotes listed in Table 1).

**Table 1:** Overarching Themes

	**OVERARCHING THEME**		
	**Helpfulness of Individual**	**Personal Contacts**	**Frequency of Contact**
**STATEMENT**	***“The falls group have been really helpful and [Falls Staff Member] in particular has been in each session” (Patient 1)**** (Falls Staff Member (Physio) placed in level 1 of the concentric circle diagram)*	***“My family and friends are the most important and helpful to me” (Patient 12)**** (Family and Friends placed on level 1 of concentric circle diagram)*	***“…these are the people I see quite regularly [pointing to centre]” (Patient 6)***
	***“The other ones are helpful, but not as much” (Patient 5)**** (Referring to those that are placed outside level 1)*	***“I rely as much as possible on my daughters” (Patient 15)**** (Daughter placed on level 1 of concentric circle diagram)*	***“I see these people often at the moment [pointing towards level 1]” (Patient 7)***
	***“These people have all been helpful day by day” (Patient 9)**** (All individuals placed on level 1 of the concentric circle diagram)*	***“I would count on my family the most if I had an emergency – that’s why my sister is right in the middle” (Patient 16)**** (Family (Sister) placed on level 1 of concentric circle diagram)*	***“…these people have all been helpful day by day” (Patient 9)**** (Individuals all placed on level 1 of concentric circle diagram)*
	***“All have helped me a lot” (Patient 4)**** (All individuals placed on level 1 of the concentric circle diagram)*		***“Just the people I see regularly really [reference to level 1]. I put [Hospital] there [level 2] because I'm not there very often…” (Patient 13)***

### Synthesised analysis of thematic and network findings

#### Healthcare professionals

Healthcare professionals described in the patient reported networks represented 47/85 (55%) of the total network—the most common. The healthcare professionals the patients mentioned were placed at different levels, more in the outer levels of the network (Fig. 2). Comments made by the patients about healthcare professionals often concerned the healthcare professionals in the FPS (as a team or as individual members). Many comments were positive, providing rationale for their inclusion:

*“The falls group have been really helpful and [Falls Staff Member] in particular has been in each session” (Patient 1) [Placed on level 1 of concentric circle diagram]*

*“The falls group has been good too” (Patient 2) [Placed on level 2.5 of concentric circle diagram]*

*“The falls team have been good encouragement and good support”* *(Patient 11) [Placed on level 1 of concentric circle diagram]*

Nevertheless, specific references to other healthcare professionals not part of the FPS were also made, including:

*“…the GP referred me here” (Patient 5) [Placed on level 1 of the concentric circle diagram]*

*“The OT who assessed my house wasn’t very effective” (Patient 11) [Placed on level 4 of the concentric circle diagram]*

*“The other people like GP and neighbours would be there to assist with my care but don't contribute as much” (Patient 16) [GP placed on level 2 of the concentric circle diagram]*

Healthcare professionals described in the healthcare professional reported networks represented 59/93 (63%) of the total network – 12 more (8% higher) than in patient reported networks. Some healthcare professionals were positioned in the centre of the network (Fig. 3), but there is greater dispersion and a greater number positioned on the outermost levels.

#### Family/friends

Family/Friends in the patient reported networks represented 23/85 (27%) of the total network individuals. Many of the Family/Friends mentioned by the patients are concentrated in the centre of the network (Fig. 2), indicating that Family/Friends are amongst those contributing the most with respect to the care and support needs of the patients. Patient comments related to their concentric circle diagram included the following:

*“…the people who care for me most would be my family” (Patient 2)*

*“My family and friends are the most important and helpful to me” (Patient 12)*

*“I would count on my family the most if I had an emergency” (Patient* *16)*

No comments mentioned the frequency of interaction, even though participants may have lived and/or socialised with Family/Friends frequently.

Family/Friends in the healthcare professional reported networks represented 27/93 (29%) of the total network—similar to patient reported networks. Though many Family/Friends are positioned in the centre of the network (Fig. 3), there is greater dispersion i.e., more positioned outside the centre of the network.

#### Group activities

‘Group Activities’ in the patient reported networks represented 7/85 (8%) of the total network. Just over half the group activities mentioned by patients are positioned at the centre of the network, indicating a high level of contribution to the current care and support of the patients.

No specific comments regarding the group activities from the patients were reported. However, some general comments were made regarding the placement of individuals by patients, which encompassed the placement of the group activities. Examples of this included:

*“All have helped me a lot” (Patient 4) (All individuals placed on level 1 of the concentric circle diagram, which included placement of a ‘Group Activity’).*

*“Just the people I see regularly really [reference to level 1] (Patient 13) (‘Group Activity’ placed in level one of the concentric circle diagram).*

‘Group Activities’ in the healthcare professional reported networks represented 5/93 (5%) of the total network, which (as in the patient reported networks) represented the third-highest category out of the five categories created, all but one being placed outside the centre of the network (Fig. 3). This contrasts with the positioning by the patients. Overall, the patients reported ‘Group Activities’ to contribute more to their care and support than did the healthcare professionals.

#### Carers/helpers

Carers/Helpers in the patient reported networks represented 6/85 (7%) of the total network (Fig. 2). The Carers/Helpers were positioned at all levels of the network. There were fewer comments about this category, the majority concerning the overall network (i.e., general comments), such as:

*“I put the ones who contribute the most here” [Points to the centre of the network] (Patient 14)*

*“All have helped me a lot” (Patient 4) [‘Carers/Helpers’ placed on level* 1 of the concentric circle diagram].

However, along with these comments, some specific ones were made describing the placement of the Carers/Helpers:

*“Social services have been rubbish” (Patient 8) [Placed on level 4 of the concentric circle diagram]*

*“[Carer/Helper Provider] I only saw once” (Patient 7) [Placed on level 4* of the concentric circle diagram]

Given these comments, it appears plausible to suggest that the placement of Carers/Helpers has been based on the helpfulness of the individual and the frequency of contact (two overarching themes identified).

‘Carer/Helper’ in the healthcare professional reported networks represented 2/93 (2%) of the total network – the fourth highest of the five categories.

The healthcare professionals positioned ‘Carers/Helpers’ outside the centre of the network in the patient perspective, where there was more diversity.

#### Other

‘Other’ in the patient reported networks represented 2/85 (2%) of the total network and were usually placed in the outer ends of the network (Fig. 2). No specific comments were made with regards to the individuals classified as ‘Other’.

The healthcare professionals who participated did not mention any individuals that would be classified as ‘Other’ in this study.

The patients mentioned ‘Building Manager’ and ‘Home Inspector’, as individuals who had provided care and support to them. As these individuals are not clinical, they would be unlikely to be recorded in a patient’s notes and may explain why they were not mentioned by the healthcare professionals.

## Discussion

This study considered the perceptions of 16 ‘patients’ who attended a FPS and those of 16 HCPs involved in their care. Although studies on frail individuals and their care and support networks have been conducted [33–36], this study is novel as it provides an insight into frail individual networks from both patient and HCP perspectives, using network analysis. Our study demonstrates the multidisciplinary nature of the care and support network, with a variety of different individuals/professions identified. It also gives an insight of HCP perceptions regarding contribution levels of different individuals involved in care and support.

This study illustrated that the variability of patient networks were similar to the perceived networks from HCPs. When the collated networks were analysed, patients and providers also had a similar overall representation of ‘Family/Friends’, though there was less variation in the reported contribution level of ‘Family/Friends’ from patients than from the HCPs. Nonetheless, the general agreement between both parties on the overall representation of the Family/friends, indicates the value that family and friends bring. Both patients and HCPs mentioned ‘Group Activities’ (e.g., FPS attendance) in the care and support networks. Given the study site, it is expected that the FPS may have been included in the care and support networks. Nevertheless, the results indicate that the contribution of the FPS with regards to care and support was enough for both parties to include in the network.

Though the ‘Health Care Professionals’ category was perceived as comprising the most to a patient’s care from both parties, an 8% difference was found. This suggests that while patients view ‘Health Care Professionals’ as high contributors to their care, other categories were also importantly noted by the patients that may have been unknown to the healthcare professionals (e.g., only patients mentioned individuals placed in ‘Other’). This finding also highlights the fact that many different individuals may form part of a care and support network.

Furthermore, the ‘patients’ placed the ‘Health Care Professionals’ closer to the centre of the level model than the HCPs, indicating higher perceived contribution levels. The finding that over double the percentage of individuals in the ‘Carers/Helper’ category were mentioned by patients versus HCPs, suggests that these individuals may not have been captured in the patient record system – or were perceived to have a higher contribution level to the patients than by the HCPs.

### Comparison with literature

Our finding that a range of different people and/or services were reported indicates the unique needs and choices of the frail individuals and is potentially evidence of holistic care and support. This finding lends support to research suggesting that the changing healthcare needs of an ageing population would be supported with holistic care approaches [37], such as consideration of an individual’s physical, social, mental and environmental circumstances. Given that frailty has been acknowledged to comprise multiple domains (i.e., not just physical and mental health domains) [13], addressing frailty with a holistic approach is a current recommendation in frail populations [38–41].

The’Group Activities’ reported may reflect the role of social or physical activities in maintaining individual’s sense of “continuity of self” [42] and is consistent with anecdotal reports from clinicians that attendees at therapeutic groups often request some form of continuing group activity. Moreover, as detailed in the introduction, social [43, 44] and physical [45, 46] activities can also prevent or lessen the progression of frailty. These falls prevention services/groups have also shown to reduce the likelihood of falls and are cost-effective in reducing admissions to hospital [47].

The prominent representation of Family/Friends in this study echoes the literature, where the value of these people has been noted [48–50], particularly on frailty progression [51, 52]. However, this finding may additionally reflect upon high numbers of informal carers caring for family [53]. Questions need to be raised to understand whether the contribution level of care and support to an individual’s network is due to preference [54], the availability of NHS care and support [55, 56] or the expense of suitable private care and support.

Given the disparity in some of the patient and HCP networks, this study could potentially signify a low level of communication between healthcare professionals, patients and family carers, as demonstrated in other settings [57, 58]. This finding is pronounced, as it may pave the way for more responsibility or collaboration if a care and support plan is produced [59]. While the logistics of involving family or friends may not always be possible, studies show that having ‘familiar’ carers or helpers can reduce the chances of illness – in addition to improving social and mental aspects of an individual [51, 60].

### Strengths and limitations

Due to the recognised population economic, ethnic and health diversity in this region, we believe the findings would be of interest to health and care settings across the UK and offer some generalisability to other regions in the UK. This study also contributes to the literature on care and support networks with respect to frail individuals.

The care and support networks of non-participants in this study may have differed to the findings evidenced in this study. Importantly, this study captures networks over a short time-frame and may not necessarily be indicative of longer term care and support networks. Moreover, it is possible that comments regarding the FPS may have resulted because of the location of the interview (at the FPS site). No participants were interviewed in their place of residence (due to the ethical and practical challenges of interviewing in a home setting [61]), which may have been easier for non-participants from the FPS.

Only patients able to understand English with full mental capacity were included. Therefore, the care and support networks of people not satisfying these criteria (yet frail) are not represented by the results in this study.

## Conclusions

This study emphasises the importance of the role of informal carers and family/friends along with healthcare professionals in the care of an individual living with frailty. While some congruence between the ‘patient’ and ‘provider’ reported networks is demonstrated, this study suggests a need for explicit discussion about care and support perceptions so that a patient centred collaborative approach is strengthened. Ultimately, the information presented in this study could be used to help frailty care and support planning and resource allocation in the community.

Further research would help confirm the reasoning behind the lower number of Carers/Helpers evidenced in this study. Moreover, given the contribution and importance of Family/Friends reported (who may be acting as informal carers), an additional study could be conducted to understand the efforts made to contact, integrate and involve these groups of people (e.g., via NHS services) in care – and to understand whether the contribution and importance of this group remains consistent over time.

## Supplementary Information

**Additional file 1.**

## Data Availability

The datasets generated and/or analysed during the current study are not publicly available due to the maintenance of confidentiality of our participants but are available from the corresponding author on reasonable request.

## Abbreviations

UK: United Kingdom
NHS: National Health Service
CLCH: Central London Community Healthcare Trust
FPS: Falls Prevention Service
SPSS: Statistical Package for the Social Sciences
HCP: Healthcare Professional
GP: General Practitioner
