# Supplementary material for: Care and support networks of community-dwelling frail individuals in North West London: a comparison of patient and healthcare workers’ perceptions
Source: BMC Geriatr. 2022 Dec 9;22:953. doi: 10.1186/s12877-022-03561-y (PMC9737751; doi:10.1186/s12877-022-03561-y)
Supplement: Supplementary file 1 — Additional file 1. [file 12877_2022_3561_MOESM1_ESM.pdf]

Appendix A

Imperial College London

NIHR CLAHRC for Northwest London

Participant Information Sheet:

Exploring the networks of individuals within a community setting

(IRAS ID: 218673, DATE: 05/05/17, VERSION: 1.1)

Investigators: Mr David Sunkersing, Professor Derek Bell, Professor Finbarr Martin, Dr Julie Reed. Department of Medicine, Imperial College London & NIHR CLAHRC NWL

Dear Participant,

Please take some time to read though the following information. If at any point you have any questions, or would like more information, please do not hesitate to ask the researcher, Mr David Sunkersing, or email at a later date at: d.sunkersing@imperial.ac.uk.

What is the purpose of the study?

This study seeks to explore the care networks of individuals within a community setting who have recently used a 'Falls Prevention' service. This study could provide useful information for healthcare professionals and influence the way care is delivered and provided for those in the community. This study is part of my final year for my PhD project at Imperial College London.

Why have I been invited?

You have been invited as you have recently used a 'Falls Prevention' service – and therefore have first-hand experience of some of the care that is delivered across central London. You may also have further experience and/or insights into other types of care or support with aspects of day-to-day living.

Do I have to take part?

Your participation is entirely voluntary. You can refuse to participate without penalty and you can stop your participation at any time. Your refusal will not impact current or future relationships with Imperial College London or NIHR CLAHRC NWL.

How will the study be conducted?

You have been invited to participate in a confidential and anonymous face-to-face interview and activity lasting around 30 minutes. If you agree to participate, a suitable time and date will be arranged prior to the end of December 2017.

This interview and activity will consist of one, single interview where a series of questions will be asked to elicit the individuals involved in your day-to-day living, or care. There will be a short activity based on these responses, whereby Post-it notes will be placed on a circle to create a visual output.

With your permission, we will also ask the healthcare professionals within the 'Falls Prevention' service/team who they believe is involved in your day-to-day living, or care – and ask them to complete the same activity.

What are the possible advantages of taking part?

You may find the study interesting and enjoy answering questions about aspects of care you have experienced. The information we get from the study will help us to understand more about the care provision and delivery in central London. These experiences and insights are valuable to us – and could help in contributing to improvements in the way care is delivered.

What are the possible disadvantages of taking part?

You may not be comfortable talking about the experiences and insights you have with your care, or aspects of your day-to-day living.

What if there is a problem?

If you are harmed by taking part in this research project, there are no special compensation arrangements. If you are harmed due to someone's negligence, then you may have grounds for a legal action. Regardless of this, if you wish to complain, or have any concerns about any aspect of the way you have been treated during the course of this study then you should immediately inform the Investigator (David Sunkersing - 020 3315 8144; d.sunkersing@imperial.ac.uk). The normal National Health Service complaints mechanisms are also available to you. If you are still not satisfied with the response, you may contact the Imperial AHSC Joint Research Compliance Office (jrco@ic.ac.uk).

The Patient Advice and Liaison Service (PALS) at Central London Community Healthcare Trust (CLCH) is also available to offer confidential advice, support and information on health-related matters (0800 368 0412; clchpals@nhs.net).

Will my taking part in the study be kept confidential?

Yes. All of the information that you give will be anonymised so that those reading reports from the research will not know who has contributed to it.

With permission, the study will additionally be audio-recorded by the researcher - on the understanding that the recordings will be kept in a secure locked cabinet at Chelsea and Westminster Hospital and destroyed after transcription.

Nobody other than the researchers will have access to the data, which will be saved securely on password protected computers and stored securely for 10 years on Imperial College London premises in accordance with the Data Protection Act 1998.

What will happen to the results of the research study?

We aim to publish the results of the study in academic papers and present at academic conferences. We also aim to engage healthcare professionals, the NHS – and the general public with the results.

You will not be identified in any report/publication.

Who is sponsoring the research?

The research is sponsored by Imperial College London.

What happens now?

Thank you for reading this information sheet and for considering taking part in this research.

If you are interested in taking part in the study, you are asked to complete the attached response slip and return it to a member of the 'Falls Prevention' team, who will contact one of the researchers. You will then be contacted to arrange a meeting at a suitable time and date (to be held at where you usually attend your 'Falls Prevention' class).

Please retain this document for your records

Response Slip

By my signature below, I confirm that I am willing to be contacted by one of the researchers about organising a suitable date and time for the interview and activity. The interview and activity will take place where you usually attend your 'Falls Prevention' class.

Please print the following information:

Name: ____

Date: ____

Contact Number: ______________________________________

Contact Email: _______________________________________

Signature: ____

Appendix B

Imperial College London

NIHR CLAHRC for Northwest London

Participant Information Sheet:

Exploring the networks of individuals within a community setting

(IRAS ID: 218673, DATE: 05/05/17, VERSION: 1.1)

Investigators: Mr David Sunkersing, Professor Derek Bell, Professor Finbarr Martin, Dr Julie Reed. Department of Medicine, Imperial College London & NIHR CLAHRC NWL

Dear Participant,

Please take some time to read though the following information. If at any point you have any questions, or would like more information, please do not hesitate to ask the researcher, Mr David Sunkersing, or email at a later date at: d.sunkersing@imperial.ac.uk.

What is the purpose of the study?

This study seeks to explore the care networks of individuals within a community setting who have recently used a 'Falls Prevention' service. This study could provide useful information for healthcare professionals and influence the way care is delivered and provided for those in the community. This study is part of my final year for my PhD project at Imperial College London.

Why have I been invited?

You have been invited as you are a member of a 'Falls Prevention' service – and therefore have first-hand knowledge and experience of some of the care that is delivered across central London. You may also have further insights into other types of care or support with aspects of day-to-day living.

Do I have to take part?

Your participation is entirely voluntary. You can refuse to participate without penalty and you can stop your participation at any time. Your refusal will not impact current or future relationships with Imperial College London or NIHR CLAHRC NWL.

How will the study be conducted?

You have been invited to participate in a confidential and anonymous face-to-face interview and activity lasting around 30 minutes. If you agree to participate, a suitable time and date will be arranged prior to the end of December 2017.

This interview and activity will consist of one interview where a series of questions will be asked to elicit the individuals involved in a patient's day-to-day living, or care. There will be a short activity based on these responses, whereby Post-it notes will be placed on a circle to create a visual output.

What are the possible advantages of taking part?

You may find the study interesting and enjoy answering questions about the different aspects of care. The information we get from the study will help us to understand more about the care provision and delivery in central London. These experiences and insights are valuable to us – and could help in contributing to improvements in the way care is delivered.

What are the possible disadvantages of taking part?

You may not be comfortable talking about the insights you have regarding care.

What if there is a problem?

If you are harmed by taking part in this research project, there are no special compensation arrangements. If you are harmed due to someone's negligence, then you may have grounds for a legal action. Regardless of this, if you wish to complain, or have any concerns about any aspect of the way you have been treated during the course of this study then you should immediately inform the Investigator (David Sunkersing - 020 3315 8144; d.sunkersing@imperial.ac.uk). The normal National Health Service complaints mechanisms are also available to you. If you are still not satisfied with the response, you may contact the Imperial AHSC Joint Research Compliance Office.

The Patient Advice and Liaison Service (PALS) at Central London Community Healthcare Trust (CLCH) is also available to offer confidential advice, support and information on health-related matters (0800 368 0412; clchpals@nhs.net).

Will my taking part in the study be kept confidential?

Yes. All of the information that you give will be anonymised so that those reading reports from the research will not know who has contributed to it.

With permission, the study will additionally be audio-recorded by the researcher - on the understanding that the recordings will be kept in a secure locked cabinet at Chelsea and Westminster Hospital and destroyed after transcription.

Nobody other than the researchers will have access to the data, which will be saved securely on password protected computers and stored securely for 10 years on Imperial College London premises in accordance with the Data Protection Act 1998.

What will happen to the results of the research study?

We aim to publish the results of the study in academic papers and present at academic conferences. We also aim to engage healthcare professionals, the NHS – and the general public with the results.

You will not be identified in any report/publication.

Who is sponsoring the research?

The research is sponsored by Imperial College London.

What happens now?

Thank you for reading this information sheet and for considering taking part in this research.

If you are interested in taking part in the study, you are asked to complete the attached response slip and return it to [the Falls Lead] who will forward it on to me. You will then be contacted to arrange a meeting at a suitable time and date (to be held at where you usually run the 'Falls Prevention' class).

Please retain this document for your records

Response Slip

By my signature below, I confirm that I am willing to be contacted by one of the researchers about organising a suitable date and time for the interview and activity. The interview and activity will take place where you usually run the 'Falls Prevention' class.

Please print the following information:

Name: ____

Date: ____

Contact Number: ______________________________________

Contact Email: _______________________________________

Signature: ____

Appendix C: Consent Form (Patients)

Imperial College London

NIHR CLAHRC for Northwest London

IRAS ID: 218673
DATE: 05/05/17
VERSION: 1.1

Consent Form

Exploring the networks of individuals within a community setting

Please read and confirm your consent to participate in this study by initialing the appropriate box(es) and signing and dating this form.

1. I understand that my participation is voluntary, and that I am free to withdraw at any time without giving any reason and without any implications for my legal rights. ☐
2. I give permission for my participatory activity sheet to be stored securely on Imperial College London premises and any other responses to be kept on a secure password protected computer server and stored for 10 years following completion of the study (all anonymised). ☐
3. I understand that anonymised quotes may be used in publications stemming from the research but not in any way that might allow for identification of individual participants. ☐
4. I understand that all personal and survey response data will be kept confidential at all times. ☐
5. I agree for my contact details to be kept on secure, password protected Imperial College London computer systems/premises, in accordance with the 1998 Data Protection Act. These will be used only to contact you and inform you of the results of the study. This information will be destroyed at the end of the study. ☐
6. I agree that my data may be accessed by responsible persons from the Sponsor, NHS Trust, or regulatory authorities, in order to check that the study has been conducted correctly. ☐
7. <u>Optional:</u> I give permission for the interview to be audio-recorded by the researcher, on the understanding that the recordings will be kept in a secure locked cabinet at Chelsea and Westminster Hospital and destroyed after transcription. ☐
8. I give permission for the healthcare professionals within the Falls Prevention team to complete an activity sheet based on their perception of my day-to-day living/care. ☐
9. A copy of this consent form will be kept for 10 years following completion of the study. ☐
10. I agree to take part in this study. ☐

Name of participant	Date	Signature
Name of researcher	Date	Signature

Appendix D: Consent Form (Healthcare Professionals)

Imperial College London

NIHR CLAHRC for Northwest London

IRAS ID: 218673
DATE: 05/05/17
VERSION: 1.1

Consent Form

Exploring the networks of individuals within a community setting

Please read and confirm your consent to participate in this study by initialing the appropriate box(es) and signing and dating this form.

1. I understand that my participation is voluntary, and that I am free to withdraw at any time without giving any reason and without any implications for my legal rights. ☐
2. I give permission for my participatory activity sheet to be stored securely on Imperial College London premises and any other responses to be kept on a secure password protected computer server and stored for 10 years following completion of the study (all anonymised). ☐
3. I understand that anonymised quotes may be used in publications stemming from the research but not in any way that might allow for identification of individual participants. ☐
4. I understand that all personal and survey response data will be kept confidential at all times. ☐
5. I agree for my contact details to be kept on secure, password protected Imperial College London computer systems/premises, in accordance with the 1998 Data Protection Act. These will be used only to contact you and inform you of the results of the study. This information will be destroyed at the end of the study. ☐
6. I agree that my data may be accessed by responsible persons from the Sponsor, NHS Trust, or regulatory authorities, in order to check that the study has been conducted correctly. ☐
7. Optional: I give permission for the interview to be audio-recorded by the researcher, on the understanding that the recordings will be kept in a secure locked cabinet at Chelsea and Westminster Hospital and destroyed after transcription. ☐
8. A copy of this consent form will be kept for 10 years following completion of the study. ☐
9. I agree to take part in this study. ☐

______________	________	________
Name of participant	Date	Signature
______________	________	________
Name of researcher	Date	Signature

Appendix E: Questions For 'Patients'

1. From time to time, you may discuss your day-to-day needs with various people in different roles. Who are the people you discuss these needs with?

2. Are any of the following people involved with your day-to-day needs:

- Doctor (GP)/Nurse/Community Pharmacist/Dietician/Physiotherapist/Occupational Therapist/Carer/Family/Friends

3. Is there anyone who helps you with the physical aspects of day-to-day living? For example, this could be someone who helps you get from one place to another or helps you with walking or mobility.

4. Who are the people you enjoy socialising with?

5. Is there anyone who checks up on your feelings, thoughts and actions or your memory?

6. Is there anyone who visits you to ensure that the inside and outside of your home is looked after? This could be someone who ensures you can easily and safely: walk around your house, get in and out of bed, see clearly due to the lighting, use the toilet and bath, reach items in the kitchen, use steps/stairs and are wearing well-fitting footwear.

7. If you had an accident, such as a fall, who would you immediately contact?

8. Do you use any resources in relation to your care needs? Some examples:

- Internet Search/Social Media Networks (e.g. Health Unlocked)/Leaflets/Books

9. How would you describe the level of contribution of this individual/resource in your care? (Participant will place Post-it note on 'Concentric Circle of Influence' diagram to signify answer).

10. How do you feel about the current diagram that has been produced. Is there anything that you would change?

Appendix F: Questions For 'Provider'

1. From time to time, [Patient X] may discuss their day-to-day needs with various people in different roles. Who are the people they discuss these needs with?

2. Are any of the following people involved with [Patient X's] day-to-day needs:

- Doctor (GP)/Nurse/Community Pharmacist/Dietician/Physiotherapist/Occupational Therapist/Carer/Family/Friends

3. Is there anyone who helps [Patient X] with the physical aspects of day-to-day living? For example, this could be someone who helps them get from one place to another or helps you with walking or mobility.

4. Who are the people [Patient X] enjoys socialising with?

5. Is there anyone who checks up on [Patient X's] feelings, thoughts and actions or their memory?

6. Is there anyone who visits [Patient X] to ensure that the inside and outside of their home is looked after? This could be someone who ensures they can easily and safely: walk around their house, get in and out of bed, see clearly due to the lighting, use the toilet and bath, reach items in the kitchen, use steps/stairs and are wearing well-fitting footwear.

7. If [Patient X] had an accident, such as a fall, who would they immediately contact?

8. Does [Patient X] use any resources in relation to their care needs? Some examples:

- Internet Search/Social Media Networks (e.g. Health Unlocked)/Leaflets/Books

9. How would you describe the level of contribution of this individual/resource in [Patient X's] care? (Participant will place Post-it note on 'Concentric Circle of Influence' diagram to signify answer).

10. How do you feel about the current diagram that has been produced. Is there anything that you would change?
